# *Kingella kingae* Keratitis in a Child with Underlying Vernal Keratoconjunctivitis

**DOI:** 10.1155/2017/1087821

**Published:** 2017-05-04

**Authors:** Salim Nurul-Laila, Khai-Siang Chai, Ahmad Tajudin Liza-Sharmini, Ismail Shatriah

**Affiliations:** ^1^Department of Ophthalmology, School of Medical Sciences, Universiti Sains Malaysia, Health Campus, 16150 Kubang Kerian, Kelantan, Malaysia; ^2^Hospital Universiti Sains Malaysia, 16150 Kubang Kerian, Kelantan, Malaysia

## Abstract

*Kingella kingae* had rarely been reported as a causative organism for corneal ulcer and had not been described before in vernal keratoconjunctivitis (VKC). Generally regarded as commensals of respiratory tract particularly in young children, it had however been isolated from the corneal ulcer scraping of both adult and children. We report a case of bacterial ulcer with isolation of* Kingella kingae* from the corneal scraping in a young child with underlying VKC.

## 1. Introduction

*Kingella kingae* is a facultative anaerobic, nonmotile, and fastidious *β*-hemolytic Gram-negative coccobacilli. It is an oxidase-positive, yielding negative catalase organism that is usually found as commensals of posterior pharynx and mouth [1]. Patients with invasive* Kingella kingae* breach the epithelium to enter the bloodstream and commonly presented with skeletal infection, bacteraemia, and endocarditis. It had been identified as an emerging pathogen amongst children, possibly due to better detection [2].

This organism had rarely been reported as a causative organism for corneal ulcer. Based on PubMed literature search, since 1992 until 2016, there were only 5 reported cases of corneal ulcer associated with* Kingella kingae* [3–5]. There was no previous report on patients with underlying vernal keratoconjunctivitis (VKC). The first case of* Kingella kingae* isolated from a corneal ulcer was reported by Mollee et al. in 1992 in Australia [3]. We describe here a case of corneal ulcer caused by* Kingella kingae* in a paediatric patient with underlying VKC.

## 2. Case Report

A 4-year-old boy who is followed up for VKC presented to the casualty clinic with right eye pain and refusal to open his right eye for 2 days. His mother noted a whitish opacity over his right eye on the day of presentation.

He had no previous history of local steroid injection for his VKC and no previous episode of shield ulcer. On his latest visit, there was compromised corneal epithelium with presence of multiple punctate epithelial erosions. However, there was no shield ulcer or corneal scar documented. His usual topical treatment consists of olopatadine twice daily and hydroxypropyl methylcellulose 6 hourly in both eyes. There was no history of recent eye trauma or foreign body that could cause corneal injury. He was not a contact lens wearer.

**Figure 1:**
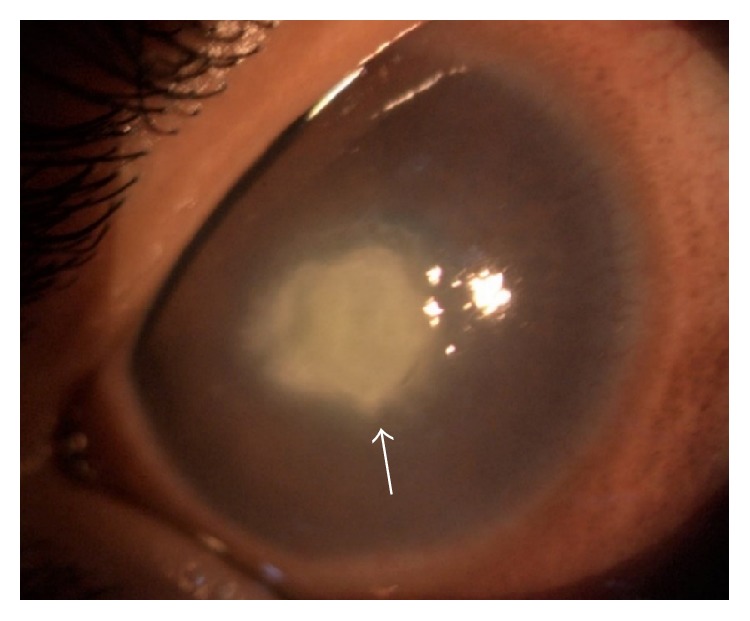
*Kingella kingae* corneal ulcer with fluffy edge and dense stromal infiltrate.

On examination, the right eye conjunctiva was hyperaemic with presence of active papillae over the eyelids. There was presence of central corneal ulcer with fluffy edge measuring 3.6 × 4.0 mm associated with dense stromal infiltrate (Figure 1). The anterior chamber showed intense inflammation with fibrin and a streak of hypopyon. His right intraocular pressure was increased to 36-37 mmHg in contrast to 20 mmHg over the left eye.

Corneal scraping was collected and sent to microbiology laboratory for Gram staining and culture. The Gram stain reviewed Gram-negative coccobacilli.* Kingella kingae* was isolated from the corneal ulcer and detected by nucleic acid amplification technique using VITEK 2 Systems Version 07.01 with very good identification and 94% probability of* Kingella kingae*.

He was admitted for intensive treatment and commenced on topical moxifloxacin (0.5%) hourly and ceftazidime (5%) hourly, along with topical intraocular pressure lowering agents: timolol maleate twice daily and dorzolamide eight hourly. The topical treatment for VKC was continued.

He showed good improvement with topical antimicrobial therapy and was discharged after seven days of treatment in the ward. Treatment was continued in tapering dosage as outpatient basis with frequent regular follow-up. On subsequent visit, his intraocular pressure normalized and his intraocular pressure lowering agents was stopped. The ulcer healed well with presence of residual central corneal scar.

## 3. Discussion

*Kingella kingae* was previously considered an organism that rarely causes diseases [1]. However, this may be attributed to the fastidious nature of the organism [6] which makes it difficult to be isolated from the affected site. With the advances of detection technique [7, 8], it has been increasingly recognized as a pathogenic organism, particularly in skeletal system infection, endocarditis, and bacteraemia [2].

*Kingella kingae* is generally regarded as a pathogen mainly amongst children [2]. However, it may also affect a broad range of age from babies to adult in cases of corneal ulcer with the youngest reported age being 11 months and the oldest being 45 years [3–5].

VKC is an allergy mediated disease that causes disruption of the ocular surface. Clinical features include itching red eye with giant papillae, Trantas dots, bulbar conjunctival pigmentation, and mucous discharge. VKC may be further complicated by development of corneal scar, shield ulcers, microbial keratitis, limbal stem cell deficiencies, and complications related to corticosteroid treatment such as cataract and glaucoma [9]. Saboo et al. described that the prevalence of microbial keratitis amongst patients with VKC is about 0.42% [9].

Table 1 summarizes published cases of corneal ulcer patients with* Kingella kingae* infection. Contact lens usage and human immunodeficiency virus (HIV) positive status had been reported [4, 5].* Kingella kingae* was isolated from a corneal ulcer in an 11-month-old baby with no ocular or systemic comorbidity [3]. Another case described by Megged et al. [5] in an 18-year-old teenager also had no other predisposing factor that could compromise the state of cornea or immune status.

Corneal ulcer caused by* Kingella kingae* responded well to topical antimicrobial therapy in all the reported cases [3–5]. The type of antimicrobial therapy used varies from aminoglycosides, cephalosporins, fluoroquinolones, and glycopeptide antibiotics. However, the patient with positive HIV infection was lost to follow-up, without definitive final outcome status [4].

Our patient has a compromised state of cornea evidenced by presence of multiple punctate epithelial erosions. The preexisting VKC resulted in unhealthy ocular surface, which may serve as a precipitating factor. The most probable mechanism by which* Kingella kingae* causes corneal ulcer is through direct spread of the organism from the mouth to contaminate hands and frequent rubbing of the itchy eye. It may also be facilitated by the compromised corneal status.

In VKC individuals, the dendritic cells which are important in initiating immune response were of a more mature phenotype, but less potent T-cell stimulators compared to their normal counterparts [10]. This may contribute to abnormal inflammatory response and increase the susceptibility to infection [10]. Punctate epithelial erosion which is a feature of VKC and indicative of desiccating stress on the cornea surface causes further disruption of corneal barrier function via T helper- (Th-) 1 and Th-17 response and involvement of interleukin- (IL-) 17 [11].

Although in most cases* Kingella kingae* infection usually runs a benign clinical course and in most cases of corneal ulcer, it healed well with antimicrobial therapy, in this case, presence of residual central corneal scar may complicate the condition with amblyopia. Thus, early detection and prompt treatment are necessary to prevent further sequelae from keratitis.

## 4. Conclusion

*Kingella kingae* is an emerging pathogen in paediatric age group that can rarely cause corneal ulcer. It generally responds well to topical antimicrobial treatment. This case report and literature review add to the current knowledge on* Kingella kingae* related corneal ulcer in paediatric population.

## Figures and Tables

**Table 1:** Reported cases of *Kingella kingae* corneal ulcer.

Number	Author & year	Age/gender	Ocular and systemic comorbidity	Clinical findings	Treatment	Outcome
(1)	Theo Mollee et al., 1992	11 months/male	Nil	Red eye, eye discharge	Gentamicin and cephalothin	Ulcer healed

(2)	Maria-Carmen Munoz-Egea et al., 2010	38 years/female	Contact lens wearer	Painful red eye for 2 days Mixed hyperaemia with paracentral corneal infiltrate measuring 1 × 1 mm	Moxifloxacin and tobramycin	Healed in 19 days

(3)	Maria-Carmen Munoz-Egea et al., 2012	45 years/male	HIV positive	Painful red eye for 2 weeks	Vancomycin and ceftazidime, then changed to gentamicin and ciprofloxacin	Unknown. Loss to follow-up

(4)	Orli Megged et al., 2013	15 years/female	Soft contact lens wearer	Painful red eye. Small corneal ulcer with grade 2 flare	Cefazoline, gentamicin	Healed in 6 weeks with faint scar and no visual impairment

(5)	Orli Megged et al., 2014	18 years/female	Nil	Painful red eye. Small 2.5 mm corneal ulcer at peripheral cornea	Cefazoline, gentamicin	Healed completely in 9 days
